# Water in Pain

**Published:** 1875-04

**Authors:** 


Water in Pain.—When will the value of water as a controller of pain be understood? There is hardly a kind of suffering known to man in which water may not be used to lessen the misery. Take a "felon"—the most terribly painful swelling which mortal ever endured—and treat it with hot water, and your pain will cease in a short time. You need not cut, nor blister, nor do anything except immerse the finger or the hand or the entire arm, in water as hot as can be borne, and keep it there until you can take it out and swing it back and forth without suffering. The hot water must be renewed as often as it cools off. The same treatment will prevent serious results in cases of puncture of the foot from rusty nails. There is nothing like it in such cases.

---

The McComber Last.—Our friends in Brooklyn, not less than those at a distance, should not forget that all first-class boots and shoes, for all ages and for both sexes, in the various fashionable styles, made on the McComber patent last and on no other, are kept for sale and made to measure by Mr. F. Edwards, 166 and 168 Atlantic Avenue, Brooklyn. These goods cost no more than the ill-formed affairs in general use, and persons once wearing them would not afterwards accept the old-fashioned kind as a gift.

---

Thousands of young men do not marry, because of their inability to meet the demands of the age; while hundreds do marry, try the experiment of high living, and sink hopelessly under a load of accumulated debt.

Give Him Light.—If a child wants a light to go to sleep by, give him one. The sort of Spartan firmness which walks off and takes away the candle and shuts all the doors between the household cheer and warm and pleasant stir of evening mirth, and leaves a little son or daughter to hide under bedclothes and get to sleep as best it can, is not at all admirable; it is after the pattern of Giant Despair, whose grim delight—confided to Diffidence, his wife—over the miseries of his wretched prisoners, always seems most inimitable,—a perfect picture of the meanness of despotism. Not that the dear mother means to be cruel when she tries this or that hardening process, and treats human nature as if it were clay, to be molded into any shape she may please. Very likely she has no idea whatever of the injury and suffering she causes, or perhaps her heart aches; but she perseveres, thinking she is doing right, and fancies she is teaching her child a lesson of bravery, when perhaps she is making a nervous, imaginative little creature, whose after life will ever bear the impress of the horrid specters its fancy called up in those moments of lonely banishment. Then give him a light, if he wants it!

---

The house is not for festivity; it is not for sleep; but the pine and the oak shall gladly descend from the mountains to uphold the roof of men as faithful and necessary as themselves; to be the shelter always open to good and true persons—a hall which shines with sincerity, brows ever tranquil, and a demeanor impossible to disconcert; whose inmates know what they want; who do not ask your house how theirs should be kept.

